# Influences on treatment-seeking and antibiotic use for common illnesses in eastern China

**DOI:** 10.1186/s12889-023-16700-w

**Published:** 2023-09-23

**Authors:** Tingting Zhang, Hanyi Lin, Xinping Zhao, Wei Wang, Fei Yan, Helen Lambert

**Affiliations:** 1 Population Health Sciences, Bristol Medical School, University of Bristol, Bristol, UK; 2 Department of Social Medicine, School of Public Health, Fudan University, Shanghai, China; 3 Key Laboratory of Health Technology Assessment of National Health Commission, School of Public Health, Fudan University, Shanghai, China

**Keywords:** Antibiotics, Severity of illness, Accessibility, Trust, Retail pharmacies, Qualitative methods

## Abstract

**Background:**

Antibiotic resistance rates remain high in China where antibiotics are widely used for common illnesses. This study aimed to investigate the influences on people’s decisions on treatment and antibiotic use for common illnesses in eastern China.

**Methods:**

Semi-structured interviews were conducted with 29 patients recruited through convenience sampling between July 2020 and January 2021 in one hospital in County A in Zhejiang Province, and one hospital and one village clinic in County B in Jiangsu Province, respectively. All interviews were audio-recorded, transcribed verbatim and thematically analysed. This study is nested in a larger interdisciplinary mixed method project and we also compared our qualitative findings with quantitative results from a household survey conducted as part of this wider project.

**Results:**

Participants’ decisions about treatment-seeking and antibiotic use for common illnesses were found to be influenced by four interactive domains. (i) Self-evaluation of illness severity: Participants tend to self-treat minor conditions with ordinary medicines first and do not resort to antibiotics unless the condition worsens or is considered inflammation- related. Visiting healthcare facilities is seen as the final option. (ii) Access to and trust in care: These treatment-seeking practices are also associated with the perception, in contrast with retail pharmacies, hospitals provide professional and trustworthy care but are difficult to access, and hence require visiting only for severe illness. (iii) Prior experience: previous medical treatment and experiences of self-medication also influence participants’ treatment decisions including the use of antibiotics. (iv) Medication characteristics: Participants view antibiotics as powerful medicines with harms and risks, requiring consumers to carefully trade off benefits and harms before use.

**Conclusions:**

People’s treatment decisions in relation to antibiotic use in eastern China are influenced by an interplay of lay conceptual models of illnesses and antibiotics and broader organisational, social, and contextual factors. Interventions focusing on individual education to incorporate biomedical knowledge into lay understandings, and reducing situational and social incentives for self-medicating with antibiotics by strengthening access to quality professional care, would be helpful in promoting antibiotic stewardship.

**Supplementary Information:**

The online version contains supplementary material available at 10.1186/s12889-023-16700-w.

## Introduction

Antimicrobial resistance (AMR) refers to the evolution of drug-resistant pathogens, which is a global threat that causes over 700,000 deaths every year and is a major economic burden worldwide [1]. Antibiotic resistance (ABR), which occurs specifically when antibiotics become ineffective against bacteria, has become a major challenge within the larger problem of AMR, and the widespread use of antibiotics is the most important factor contributing to ABR [2–4]. Although AMR threatens all countries, it has a disproportionate impact in low and middle-income countries (LMICs), including China, where the level of overuse of antibiotics is high and there are high resistance rates to common antibiotics [5, 6]. A series of national actions to contain AMR have been introduced by China government since 2011, with the focus progressing from antibiotic use in clinical settings only to combating AMR from a one-health perspective. Some remarkable achievements have been obtained, such as the decrease of antimicrobial prescriptions for both hospitalised patients and outpatients between 2011 and 2016 after the release of the three-year ‘*National Special Campaign for the Clinical Use of Antibiotics*’ in 2011 [7]. Evidence from individual hospitals also showed the total resistance rate declining by 5.3% and culture positivity rates by 9.8% from 2015 to 2018 with the introduction of ‘*National Action Plan to Contain Antimicrobial Resistance (2016–2020)*’ [8]. However, ABR rates in China still remain high. For instance, the proportion of methicillin-resistant Staphylococcus aureus (MRSA), third-generation cephalosporin resistant Escherichia coli (3GCREC) and carbapenem resistant Acinetobacter baumannii (CRAB) in China in 2017 were 32.2%, 54.2% and 56.1%, respectively, which was almost twice as high as in Europe (16.9%, 14.9% and 33.4%) [7, 9]. The use of other sources to obtain antibiotics without a medical prescription are popular in China; a WHO multi-country public awareness survey reported that China had the highest proportions of accessing antibiotic online (5%) and obtaining from family or friends (4%) among six high income countries (HICs) and six LMICs included in the study, where these figures in other countries are 2% or 1% [10].

As antibiotics have been widely used for common illnesses such as acute respiratory tract infections in China, an understanding of the way that people seek care and treatment for common illnesses and the factors influencing their care seeking are therefore important. Many quantitative studies have done focusing on various groups of users’ (parents, adults or university students) treatment decisions and antibiotic use. They identified practices of self-medication with antibiotics and requiring antibiotics during consultation when participants have minor symptoms, and factors influencing their practices including knowledge and attitudes towards the illness (e.g. not knowing that the common cold is self-limiting), symptoms, and treatment options (e.g. keeping antibiotics at home) [11–15]. However, these practices and influencing factors can be heavily influenced by socioecological context. One study suggests that while in Western societies illness is often explained through a biomedical lens, a combination of beliefs including fatalistic, situational and biomedical beliefs is more commonly found in traditional societies to understand illness [16].

China’s health system, consisting of hospitals and primary health care, also has some characteristics that are distinct from other settings. In contrast with the referral system in HICs like the UK, both urban and rural patients can freely choose either hospital outpatient clinics or primary health care facilities as the first visit for healthcare services for minor symptoms [17]. Although primary health care is growing rapidly and has substantially contributed to disease burden reduction in China, the quality of services provided by hospitals is more highly valued and the proportion of services provided by hospitals is still substantial [18, 19]. In 2021, hospitals provided services for 3.9 billion outpatient and emergency visits, the amount is still very similar to the services provided by primary health care (around 4.3 billion outpatient and emergency visits) [20]. In keeping with hospitals being the most well-recognised form of healthcare, the public in China tend to refer to all types of health facility that provide clinical care as ‘hospitals’. China also has a strong retail pharmacy sector that compensates for the weak primary health care system. It is theoretically illegal to dispense prescribed medicines, including antibiotics, without prescriptions in China since 2000, with enforcement of regulations being strengthened gradually, but such practices remain common in China. Although licensed pharmacists are required to be present in pharmacies to give advice, the proportion of pharmacies having a licensed pharmacist on duty was still under 50% in 2017 [21].

The aim of this study was to investigate how patients make decisions on treatments, including self-medication with antibiotics, for common illnesses, and to understand the influences on these decisions within the local context from a qualitative perspective, to provides insights into the rationales driving antibiotic use and ABR in eastern China.

## Methods

### Study design

Our study is nested in a larger interdisciplinary mixed method project to investigate antibiotics in both the environment and human health in eastern China that has six key components, including (i) biological and environmental sampling; (ii) ABR genes and transmission; (iii) chemical analysis of antibiotics in the environment and urine; (iv) structured household survey; (v) medicine diary and electronic medicine record; (vi) in-depth interviews and community focus groups [22]. This paper mainly draws on data from the in-depth interviews. The research team conducted in-depth interviews with stakeholders in both human health (professionals in health facilities and retail pharmacies, health authority officials and patients) and agriculture (farmers, government agriculture officials and veterinary drug sellers), and this paper reports findings specifically from patients. It also uses these qualitative findings to further explore quantitative data from the household survey that described patterns and determinants of antibiotic use behaviours among residents in the same study areas; quantitative findings have been published separately with detailed methods described [23].

### Study area and recruitment

The qualitative stakeholder interviews were carried out in two counties in eastern China that are well-recognised as a hotspot for antibiotic consumption and resistance. County A is in a relatively rural setting in Zhejiang Province while County B is located near a large municipality of Jiangsu Province with higher accessibility to healthcare services. By the end of 2020, the household populations of County A and County B were 443,000 and 993,400, and the county’s GDP per capita is 122,787 and 145,215 RMB yuan (17,190 and 20,330 USD), respectively (1 RMB≈0.14 USD).

Two county hospitals and one village clinic at primary care level were approached in counties A and B. After obtaining consent from the three healthcare facilities, investigators attended these facilities to recruit patients for interviews. Patients at outpatient clinics, in the laboratory testing department, and the waiting area of hospital pharmacy of these three healthcare facilities were invited to participate in this study. Participants were recruited using the convenience sampling method but were purposively selected in order to obtain a maximum variation sample (a diverse range of sociodemographic characteristics). The interview sample size was determined on the basis of information saturation, and patients were continuously sampled until no new information could be obtained. 29 patients were eventually recruited.

### Data collection

Semi-structured interviews were conducted by four researchers (FY, WW, ZD, CM) in Mandarin. FY and WW are experience qualitative experts and are faculties in university, and ZD and CM are graduate students who have received essential qualitative related training. One-to-one interviews were conducted in person on site in a quiet place after participants agreed to take part and recorded using an audio-recorder. Each interview session lasted approximately 30 min. Interviews were conducted between July 2020 and January 2021.

Interviews were guided by the topic guide that explored participants views and experiences of antibiotic use. The topic guide covered demographic characteristics of participants, participants’ knowledge and awareness of antibiotics, participants’ attitudes to antibiotics, and their antibiotic use experiences. More specific questions were set under each theme and there were in total 18 questions (topic guide is provided as supplementary material).

### Data analysis

All interviews were transcribed verbatim in Mandarin and thematically analysed in NVivo 12 Pro. One researcher (TZ) predominantly analysed the interviews with patients by reading and parallelly coding a sub-sample of patient transcripts that captured participants’ views and experiences and assigning initial codes to the data with another researcher (HL); inconsistencies were discussed and resolved with wider team. Codes that recurred within and across participants were allocated into conceptual categories and themes. An initial coding framework was then developed, and the remaining transcripts were indexed using this framework with refinements where necessary. Common codes and categories emerged across transcripts, indicating data saturation.

In addition, TZ and XZ reviewed and compared the household survey questionnaire and database with the initial coding frame, and XZ conducted further analysis on the survey data based on the initial coding frame to generate results that could be compared with the qualitative findings.

## Results

Our qualitative sample consisted of 29 patients from the same counties in which the household survey was conducted. Participant characteristics are presented in Table 1. Two-thirds of the participants were female. The majority were educated to college / undergraduate level and under 60 years old. Most of participants were covered by basic medical insurance programmes, including 12 participants covered by basic medical insurance programme for urban employed (BMIUE) and 13 covered by basic medical insurance programme for urban and rural residents (BMIURR).

**Table 1:** Participant characteristics

		**County A**	**County B**
**Total number**		17	12
**Gender**	Female	12	7
	Male	5	5
**Age** ^**a**^	< 30	1	4
	30–39	4	2
	40–49	2	4
	50–59	4	1
	> = 60	2	1
**Education** ^**a**^	Primary School	0	1
	Secondary School	5	2
	Technical high school	3	1
	College / Undergraduate	4	7
	Postgraduate	0	1
**Occupation**	Employed by another person or unit	10	6
	Running an individual or private business: self-employment	4	0
	Farming	0	1
	casual work	1	3
	Retirement	2	1
	Student	0	1
**Medical insurance** ^**a**^	basic medical insurance program for urban employed (BMIUE)	9	3
	basic medical insurance program for urban and rural residents (BMIURR)	7	6
	No health insurance	1	1

^a^Missing information: 4 patients in County A missed age information, 5 patients in County A missed education information, and 2 patients from County B missed medical insurance information

Data from the household survey, including 1,379 participants from two counties, showed that the majority (59.9% of participants) obtained antibiotics from healthcare facilities with a prescription, while 17.7% of participants obtained antibiotics from pharmacies and 22.4% obtained them from other sources such as family and friends [23]. The qualitative results presented here regarding influences on patients’ treatment decisions and the use of antibiotics for common illnesses are organised into four broad themes, which interacted with each other: perceived severity of the illnesses or symptoms; a balance between trust and accessibility among healthcare facilities; past experiences; and antibiotics as powerful medicines with harms and risks.

### Perceived severity of illness

Patients described the severity of their illness and how the perceived severity influenced their decisions on the level of healthcare to be accessed. Self-evaluation of the severity of illness was a common practice and some conditions, such as cold, were perceived as not serious; many patients described how they would usually use home remedies or take some ordinary medicines that they prepared at home to treat these illnesses, and were very confident that there is no need to go to the hospital.

“[Antibiotics] will not be used for mild cold, and [medicines] to clear heat and toxin and cough syrups will be used in general.” (0111DQH3F3, 25 years old, no medical insurance)

“This means, if the symptoms are not serious, I will be surely not visiting the hospital.” (0111DQH3F2, 55 years old, BMIURR)

“We will not go to the hospital if it’s not serious, that would be fine for taking [some medicines] prepared at home.” (0117HMH3F2, 49 years old, BMIURR)

Antibiotics were described by most patients either as “*anti-inflammatory medicines*” (xiaoyan yao), or by the generic name such as cephalosporin or amoxicillin. In contrast with ordinary medicines, most patients said they would not self-medicate with antibiotics until they felt that the illness had got worse or lasted longer than expected:

“Cephalosporin, possibly when [the illness] lasts for one or two weeks, possibly taking them when not recovering after a long time.” (0714DQH3F2, 35 years old, BMIUE)

“To be honest, it is absolutely better to less frequently use this medicine [anti-inflammatory medicine].” (0116HMH3F4, 65 years old, BMIURR)

Patients who described using antibiotics in this way said they would medicat themselves with antibiotics when they felt there is a particular inflammation or had identified inflammation-related symptoms, such as sore throat or fever. This is supported by the survey data that skin / wound infection are the most common conditions (52.8%) that participants reported as needing antibiotic treatment. 41.7% and 41.1% of participants reported using antibiotics for sore throat and fever, respectively, while fewer participants would use antibiotics for more mild symptoms such as headaches (26.8%) and body aches (21.8%).

“Since cold means no inflammation, I don’t need to take [cephalosporin]. … In general, anti-inflammatory medicines will definitely not be taken if there is no inflammation.” (0714DQH3F5, 16 years old, BMIUE)

“If myself feel having temperature or inflammation, such as throat inflammation or others, I will take anti-inflammation medicines. … For example, I do take anti-inflammatory drugs, such as amoxicillin, if I feel a fever, or if I have an inflammation of the throat.” (0111DQH3F3, 25 years old, no medical insurance)

“R: For some common cold, do you hope doctor to prescribe anti-inflammatory medicines to you? Or feel like taking them as less as possible?

P: As less as possible, and not taking them if you can, this is my thought. … If you have temperature then you can take a little bit, that would be fine.” (0116HMH3F5, 41, BMIURR)

For visiting healthcare facilities, most patients said that they will seek a consultation only if the illness is serious and their self-medication practices cannot control it: “*Generally if it’s not serious, I usually stay at home and drink hot water, and then take a bit cold medicines. I will go to hospital for examination if it’s serious. … Sometimes it may be very serious and taking medicine are not effective anymore, you must go to the hospital for either hanging water* [intravenous antibiotic drip] *or something else.*” (0116HMH3F1, 27 years old, N/A).

While some patients reported visiting hospital to have a consultation or examination, some only expected treatment to control and provide relief from the illness and explained their decision to attend a healthcare facility as occurring when, “*medicines from retail pharmacies didn’t work and then you have to go to hospital to obtain their medicines*” (0111DQH3F2, 55 years old, BMIURR). This is more commonly found among aged people. For instance, hanging water, which is intravenous antibiotic drip that can only be obtained from healthcare facilities, was mentioned as the only reason by some aged patients for going to hospital:

“Cough, if taking medicines will not be effective, then going for hanging water.” (0117HMH3F1, 42 years old, N/A)

“[The development of] cold is a process and we start from taking medicines. Fever needs to have hanging water, while cold and runny nose don’t need to have that.” (0712DQH1F2, 51 years old, BMIUE)

### A balance between trust and accessibility

A further reason that patients would not see a doctor for illnesses that they perceived as mild is the difficulty in accessing healthcare facilities. Patients reported three types of concern about accessing healthcare facilities: (i) the need to travel outside their locality and associated difficulties in finding transport, (ii) time constraints including long waiting times and not having time to visit during the working day, and (iii) financial constraints that related to both cost of treatment and the opportunity cost such as travel costs.

“Headache or temperature, which look like normal cold, I will go to retail pharmacies and buy some medicine; after all, you need to wait for a long time when visiting hospitals.” (0111DQH3F2, 55 years old, BMIURR)

“*When feeling unwell and inconvenience to go to hospitals, like us* [need to] *travel from village to urban areas, it’s even inconvenient to find transport to hospital, so we have to solve them by ourselves that buy some medicines from retail pharmacies, and visit hospitals if there are no other ways, it’s so inconvenient.*” (0117HMH3F1, 42 years old, N/A) 

In contrast, one patient said that she usually visited hospital rather than self-medicated with antibiotics for common cold or cough because she can access treatment easily through her personal network:

“P: No [I will not self-medicate with antibiotics for common cold or cough], we will visit hospital

R: So you visiting hospital because you want doctors to prescribe antibiotics to you?

P: Yes, Since I have a best friend who is a doctor, I will directly come and find her.” (0713DQH3F1, N/A, BMIURR)

The financial constraints are particularly mentioned by a participant who works as a migrant worker in study place and joins the BMIURR at his hometown: “*Medicines there* [in hospital] *are too expensive; it is not about* [unwilling to] *obtain* [medicines from hospitals*], it is about cannot afford.*” (0712DQH1F1, 35 years old, BMIURR).

Most patients trusted the professionalism of hospitals. There was only one patient who criticised the lack of quality attention to patients and found the process of diagnosis and treatment problematic: “*you see our general public cannot understand these, but the doctors just simply communicate with you and prescribe either colour ultrasound or X-ray, and look at the results, that’s it. They then prescribe a lot of medicines for you, and they do not work, I always met this situation.*” (0116HMH3F1, 27 years old, N/A).

Although the majority mentioned they had previously purchased medicines from retail pharmacies since it’s convenient to access and prescriptions are not mandatory when purchasing prescribed medicines in some places, some patients preferred not to do this. Retail pharmacies were recognised to be money-making businesses and some patients did not trust them. They believed that medicines sold in retail pharmacies and hospitals could be different, even if they had the same name, and considered those from retail pharmacies as less effective.

“I have not [purchase medicines from retail pharmacies] and don’t trust them. For medicines, I will trust those from hospitals. I don’t trust medicines outside [of hospitals], the procurement sources are different, so I don’t purchase medicines outside [of hospitals].” (0117HMH3F3, 57 years old, BMIURR)

“The kind of cephalosporin in the hospital, and the cephalosporin in the retail pharmacy, should not be the same cephalosporin, is it? Maybe the quality of those from the retail pharmacy is not very…, it seems that the anti-inflammatory drugs prepared in the hospital may be better. For example, if you have any symptoms, you may be relieved by taking that medicine [from the hospital], but medicines from the retail pharmacy will not be very effective.” (0111DQH3F2, 55 years old, BMIURR)

### Prior experience and health information

Past sickness experiences and health information previously obtained from health professionals influenced patients’ help-seeking practices. In the survey, over one third (33.7%) of participants agreed with the statement that people could purchase or request the same antibiotics from doctors if they experienced the same symptoms as previously and antibiotics were effective. Six interviewed patients similarly reported such practices.

In the interviews, many patients reported that they had learned which medicines were prescribed by a doctor for particular illnesses/symptoms when they initially sought help at healthcare facilities, and they then self-medicated with the same medicines when they or their family members experienced similar illnesses/symptoms. Successful experiences with self-medication encouraged them to repeat this practice.

“Generally speaking, I know what medicines hospitals will prescribe, so I will first self-medicate these medicines to him [patient’s child] if he had a bad cough or fever … like for serious cough, fever, or having some inflammation, they [hospitals] will surely prescribe [these medicines], almost every time.” (0116HMH3F3, 31 years old, BMIUE)

Likewise, many informants knew the effectiveness of antibiotics from either previous doctor’s treatment or successful experience of self-medication, which informed their practices for recurring illnesses or symptoms.

“At past time when you went to a hospital, doctors would prescribe medicines as long as you were unwell, not prescribe medicines, they would give a hanging water as long as you were unwell, to make you recover as soon as possible.” (0111DQH3F6, 44 years old, BMIUE)

“Yes, we will take leftover [anti-inflammatory medicines] if we have at home because they are effective, it’s okay to take them if they are effective.” (0116HMH3F1, 27 years old, N/A)

### Viewing antibiotics as powerful medicines with harms and risks

Some patients described their desire to obtain relief from symptoms and control the harms of illness, and the need to go back to normal life quickly. Accordingly they highly valued antibiotics as effective and able to provide fast relief.

“Sometime when children got ill, it could influence their study. So we thought that hanging water can be recover quicker.” (0111DQH3F6, 44 years old, BMIUE)

“The effectiveness of anti-inflammatory medicines is relatively straightforward, unlike other Chinese patent medicines which become effective slowly.” (0116HMH3F3, 31 years old, BMIUE)

Despite the benefits, most patients also expressed concern about the harms of antibiotics, relating to a broader understanding of medicines as inherently toxic: “*As a medicine, it is somewhat toxic* (*shi yao san fen du*)” (0714DQH3F1, N/A, BMIURR). Patients believed that it was generally bad for the body to take too many antibiotics or use them for a long time. Some patients reported side effects as their main concern in using antibiotics, but only two informants gave a detailed description on what those side effects were.

“R: What do you think about antibiotics?

P: *Just feel it’s bad for body if taking too many*.” (0111DQH3F8, 41 years old, BMIURR)

“Not very specific thing [about use of antibiotics], but I know it cannot be taken for too long time, which is not good for body.” (0111DQH3F5, 30 years old, BMIURR)

“Like there may be side effects sort of things, antibiotics may have side effects like decreasing the immune function, should have these kinds of side effects.” (0111DQH3F2, 55 years old, BMIURR)

“Medicines must have side effects, all medicines have to be metabolized and excreted by the liver and kidneys, so I am opposed to it [taking medicine]. … For antibiotics, my stomach is not very good, and my gallbladder is also not very good. For things like cephalosporins, I sometimes seem to have gastrointestinal reactions after taking them, and I also don’t recommend my family members to use them.” (0117HMH3F3, 57 years old, BMIURR)

Patients therefore traded off benefits and harms of antibiotics and tried not to use them if possible: “*Now I feel antibiotics should be used as less as possible and even not use them. To use them if there is no choice*” (0111DQH3F5, 30 years old, BMIURR). One common way to do this, as described above, is that patients would not take antibiotics at the beginning of an illness. Preference for traditional Chinese medicine was also associated with reduced willingness to use antibiotics:

“In my family, including both adults and children, we [are] all resistant to using antibiotics. … Our family respects traditional Chinese medicine culture and rejects Western medicine, [we only use Western medicine] unless there is no choice. So in general we use Traditional Chinese medicine treatment for both adults and children in our family.” (0111DQH3F1, 56 years old, BMIUE)

When need outweighed potential harms, patients then took antibiotics but stopped using them as soon as symptoms disappeared to avoid taking too many antibiotics:

“I will stop taking medicine when I feel better, it’s not good to take too many medicines. … Yes, anti-inflammatory medicines cannot be taken too many, I will stop medicating the child once he/she looks good.” (0116HMH3F1, 27 years old, N/A)

## Discussion

A separately published paper presenting findings from the household survey component of our project found that obtaining over-the-counter antibiotics from retail pharmacies was positively associated with *both* low level of antibiotic knowledge *and* high education level [23]. A similar paradox has been reported by a study of Chinese university students, which observed a significantly higher rate of self-medication with antibiotics among medical students and identified prior knowledge of antibiotics as a risk factor for self-medication with antibiotics [24]. These findings suggest that behaviours with respect to antibiotic use and access are complex and cannot necessarily be modified simply by increasing knowledge. Our qualitative findings further illuminate this by showing how treatment decisions are made under multiple interacting influences of perceived illness severity, prior experiences, views on medicines and organisational influences related to healthcare facilities.

Our study finds that patients’ help-seeking practices are mainly based on the progression and perceived severity of symptoms, which relate to managing symptoms and returning to normal activities, but patients rarely considered a need to diagnose the condition underlying their symptoms or expressed any aetiology-related concerns, particularly for common illnesses. Self-assessment of the severity and perceived threat from the illness in deciding to seek treatment are widespread among residents across LMICs, in part due to the financial and time constraints in getting access to healthcare services such that residents had to judge whether the illness was severe enough to warrant these costs [25–30]. Patients in our qualitative interviews expressed their desire to recover and only aimed to receive specific treatments that are available in the clinics (e.g. intravenous drip) when visiting healthcare facilities, without concerns about the causes of symptoms. In Vietnam, it was also found that people frequently self-prescribed antibiotics based on symptoms but less commonly self-diagnosed the causes among mild illnesses, and perceived little difference between visiting health facilities and pharmacies [31]. As medical expertise is the distinctive difference between self-medication/purchasing medicines from retail pharmacies and professional healthcare services in health facilities, as long as people are mainly seeking symptom relief and do not consider medical consultation necessary, self-medication logically becomes the first resort, particularly for mild illness. This may also account for practices of self-medication with antibiotics regardless of whether bacterial or viral infections were involved, since illness was mainly understood through symptoms and bodily experiences rather than according to aetiology, and antibiotics’ effectiveness and power to provide a quick relief on symptoms were appreciated, based on past experiences [14, 32, 33].

Self-medication with antibiotics purchased from retail outlets without a prescription has been reported throughout LMICs [25, 34–37]. As with our qualitative findings in China, common reasons relate to purchasing of over-the-counter antibiotics being easy and convenient from retail pharmacies, in contrast with difficulties in getting access to healthcare facilities and sometimes low quality of clinical services [27, 35, 38]. The evidence from our household survey supports this by finding associations between obtaining antibiotics from retail pharmacies, being blue-collar workers (aOR 1.51, 95% CI: 1.01–2.26), and low (< = 10,000 RMB) annual household income (aOR 1.91, 95% CI: 1.34–2.73), suggesting the presence of financial constraints in access to healthcare services [23]. Our qualitative study further explained that the financial constraints could be related to insurance scheme that, despite various policies and changes, for migrants and indeed anyone seeking care outside their ‘hometown’ it is more expensive or inconvenient. The ‘long-distance transaction’, which allows the medical cost generated outside people’s hometown health facilities to be covered by basic medical insurance scheme, are only effective in certain selected health facilities, and migrants still have to pay out of pocket initially and then travel hometown to submit the receipts and get reimbursement. In our previous study, we also observed the proportion of insurance reimbursement gets lower if people are outside the home region/province.

Also, in keeping with other studies, prior successful experiences on the use of antibiotics would confirm their effectiveness and power and contribute to people seeking out antibiotics, including those without prescriptions, when similar symptoms recur [38, 39]. Whereas people in some LMICs treated pharmacists and drug sellers as doctors and asked for their advice on treatment [25, 33, 40, 41], in our interviews, patients’ views on the trustworthiness of retail pharmacies was predominantly product-oriented, relating to the perceived lower effectiveness of medicines sold in these outlets. In contrast with studies in other Asian countries which found that people valued the quality of medicines purchased from retail outlets more highly than free medicines from public health facilities [28, 42], concerns about the quality and effectiveness of medicines from retail pharmacies were frequently mentioned by patients in our study, which led them to trust retail pharmacies less and prefer not to use them. The household survey also showed that the majority of residents obtained antibiotics from healthcare facilities with a prescription. Therefore, despite facilitators such as the convenience and accessibility of retail pharmacies and previous successful self-medication experiences that could encourage purchasing, some patients may be disinclined to purchase antibiotics over the counter due to a lack of confidence in the quality of medicines sold in these outlets.

Antibiotics are understood as strong and powerful medicines among patients in our study, in keeping with views on antibiotics in other LMIC settings [27]. However, in contrast with the exclusively positive views on antibiotics reported from some studies in other countries [25, 43] and with older practices of using antibiotics for prophylaxis or as long as feeling unwell in China [44], our study finds that most patients are concerned about the harms of antibiotics as well as their effectiveness and had made efforts to reduce usage, such as delaying the initiation of antibiotics for common illness. These changes probably relate to the influence of AMR public information and education campaigns over recent years in China, as well as the increased pathways to get access to health information, including the trend that young adults in China and worldwide seek health information and antibiotic related information from Internet [45–47]. However, knowledge delivery models that aim simply to improve public awareness of AMR without considering non-biomedical drivers of health behaviours can be misinterpreted and have unforeseen adverse consequences [48, 49]. Our findings suggest that AMR campaigns that focus on public education to distinguish the pathogenic causes of illnesses (bacteria or virus) may not translate effectively into reduced use of antibiotics for viral infections since such explanations do not resonate with how most people understand illness. In addition, patients’ understandings of harms related to antibiotics were relatively vague and focused more on antibiotics’ chemical toxicity related to the quantity of consumption rather than the appropriateness of antibiotic use for specific infections. It has been reported that some public will not critically appraising the reliability of information from Internet[47]. These perceptions lead to some unforeseen practices such as shortening the course of antibiotic treatment in order to reduce the amount of antibiotics consumed to mitigate potential harm. Although new evidence and medical opinion have determined that the traditional advice to ‘complete the course’ does not prevent antibiotic resistance and that stopping antibiotics when recovered is advisable for individual patients [50], our participants’ early stopping practices did not arise from current China AMR education content. In fact, their practices contradicted the information from recent ‘*World Antibiotic Awareness Week*’ campaigns in China that advised to complete the course even after feeling better [51].

Although the number of patients participating in study interviews was limited, participants were recruited from different clinical settings in both rural and more metropolitan areas and included a diverse range of demographic characteristics. Their views add an important perspective on how people make decisions to use antibiotics and provide explanations for the household survey results. Our study only includes outpatients with higher educational background in health facilities without pharmacy customers or health residents, whose treatment decisions related to antibiotic use or views on obtaining health care might be different from participants who were attending health facilitiesAlthough the pattern of views and practices among interviewed patients is consistent with the findings from our household survey focusing on healthy residents, future qualitative studies should include broader populations, such as pharmacy customers, to better understand how decisions on antibiotic self-treatment are made. Furthermore, our study did not specifically target the elderly population, with only three patients being over 60 years old. Our household survey indicated a significant difference in practices of obtaining antibiotics from retail pharmacies between the age group over 65 and the group between 15–44. Moreover, our previous studies suggested elderly population living in rural areas with a lower educational level holds different views and attitudes towards treatment decisions and antibiotic use. As a result, future qualitative studies could focus on the elderly group and aim to understand their perspectives and practices regarding common illness and antibiotic use decision.

## Conclusions

Our study has provided detailed evidence of influences on patients’ treatment decisions in relation to antibiotic treatment in eastern China. Members of the public’s practices of antibiotic use relate to their understandings of illness and antibiotics through bodily and lived experiences within their local social context. Community interventions, including AMR information and education campaigns, should therefore focus on how to incorporate relevant information into local knowledge and lay understandings of illnesses among residents and consider all possible communication strategies to ensure the appropriate delivery. It is also worthwhile to consider how to break the positive feedback loop of self-medication with antibiotics and perceived recovery from illnesses when designing community interventions. As suggested by Mckinn et al., interventions can pose appropriate alternatives to antibiotics and emphasise the same qualities, such as effectiveness and strength, of alternatives valued in antibiotics. In addition, progress in universal health coverage and basic health insurance schemes should ensure equitable access particularly for migrants and high quality health services for all. This will help weaken the attraction of retail outlets and antibiotics that provide a ‘quick fix’ to compensate for difficult access to healthcare services.

### Supplementary Information

**Additional file 1.** Interview guidelines with key stakeholders.

## Data Availability

Stakeholder interview data will be made available through the University of Bristol data repository (https://data.bris.ac.uk/data/) on an open access basis within one year of the end date of the project (31^st^ March 2023). Requests to access the data should be directed to the authors who will provide a link without undue reservation. The household survey data may be made available upon request from the corresponding authors.

## Abbreviations

ABR: Antibiotic resistance
AMR: Antimicrobial resistance
HIC: High income country
LMIC: Low and middle-income country
