# Supplementary material for: Influences on treatment-seeking and antibiotic use for common illnesses in eastern China
Source: BMC Public Health. 2023 Sep 23;23:1849. doi: 10.1186/s12889-023-16700-w (PMC10517519; doi:10.1186/s12889-023-16700-w)
Supplement: Supplementary file 1 — Additional file 1. Interview guidelines with key stakeholders. [file 12889_2023_16700_MOESM1_ESM.docx]

**Interview guidelines with key stakeholders**

**Health administration department**

**Administrative staff**

1. **Interview guidelines with administrators in health facilities**

**(departments related to application and administration of antibacterial drugs: medical administration division, infectious disease division and so on)**

**The material needs to be collected:**

1. Regulatory documents related to usage of AB and AMR
2. **Basic information**
   1. Could you make a self-introduction?

**Probe:**

- age, gender, educational level
- department/division, administrative post, clinical speciality, professional ranks and titles, length of service (length of service of current position)

1. **Basic characteristic of the health facility**
   1. Could you introduce us your health facility?

**Probe:**

- level of the health facility (eg: health center/county hospital…)
- coverage of areas and population
- patients’ payment methods (eg: insured/self-payed… and their proportion)
  1. What are the main types of services provided by your facility?

**Probe:**

- surgery/prescription/public health, etc.

what kind of surgery?

- numbers of inpatients and outpatients per month/day?
  1. What are the main sources of revenues for your facility?

**Probe:**

- Government budget/service/drug income etc.
  1. What are the main sources of incomes for the doctors of the department?

**Probe:**

- Basic salary/performance according to prescription and physical examination, etc.
  1. What are the measurement and evaluation of services performance of the doctors in your health facility?

**Probe:**

- Who or which department in charge of evaluation?
- What are the measurement indicators: number of patient consulted/number of surgery/medical service fee/drug expenses/patient satisfaction?
- Any reward and punishment?

1. **Administration of usage of AB in the health facility**
   1. What is your understanding on current situation of usage of AB in China?

**Probe:**

- do you know any policies or regulations on usage of AB in China?

Eg: *Guiding Principles of Clinical Use of Antibiotics*, *Measures for the administration of clinical application of antibiotics*, etc.

- 1. Can you tell me about any policies of administration on AB usage and surveillance of AMR in China that are relevant to you?
  2. Can you explain who or which institution oversees administration on AB usage and surveillance of AMR in China?

**Probe:**

- Eg: Department of Hospital Service/National Medical Products Administration (NMPA), etc.

*（Since 1-Sep, 2018, CFDA (China Food and Drug Administration) has changed its name to NMPA (National Medical Product Administration）*

- What are their main duties/functions?
- Their surveillance mechanism?
  1. Has your health facility joined the Center for Antibacterial Surveillance system (CAS)?

**Probe:**

- If added,

how does your facility report data to CAS?

what data be reported to CAS by your facility?

what do you think of the quality of the data reported by your facility?

- If not,

do you think your facility should join CAS? Why?

- 1. What are the main regulations and its process of administration on usage of AB in your health facility?

**Probe:**

- management of antimicrobial drug catalogue, surveillance system, prescription review/function of pharmacy staff, etc.
- Who administrate the usage of AB?
- Who evaluate the AB prescription?
  1. Can you specify the detailed procedure on surveillance of usage of AB in your health facility?

**Probe:**

- Reporting system/prescription review, etc.
  1. What do you think of the effectiveness of those regulations?
  2. What kinds of training has been provided to the health professional?

**Probe:**

- the training format: lecture/exam/conference on AU, etc.
- whom and the frequency of these trainings
  1. Any incentives or punish policies on usage of AB in your health facility?

**Probe:**

- Eg: the lower usage rate, the higher salary/deprivation of prescription
- Does the usage of AB influence incomes for the doctors?
  1. Does TCM prescription influence the AB prescription?

**Probe:**

- Prescription proportion/Drug income, etc.
  1. How about the usage of AB among different departments in your health facility?

**Probe:**

- prescription for treatment & prevention
- the most and the least usage department
- Which department controls usage of AB the best? And why?
- Which department controls usage of AB the worst? And why?
  1. What do you think the root cause of the irrational use of AB?

**Probe:**

- lack of knowledge/ prescription habit learned from senior doctors/need extra income to compensate the low salary/ patients’ expectation and stress/ over-marketing of pharmaceutical companies
  1. Has your health facility set up any management goal or performance indicator regarding usage of AB?

**Probe:**

- **If yes,** what’s the goal & indicator system & data source?
  1. Any challenges on administrate usage of AB in your facility?
  2. In your opinion, do other types of health policies cast any impacts on the administration and usage of AB in China?

**Probe:**

- Eg: medical insurance policy/ essential medicine policy, etc.
  1. Compared with other health facility at the same level, what do you think are the characteristics and innovations of the management of usage of AB in your facility?

**Probe:**

- Any successful experience?
  1. Do you have any suggestions on improving current measurements of administration on usage of AB?

**Probe:**

- System/ training, etc.

1. **Surveillance of AMR in your health facility**
   1. Has your health facility joined the CARSS system?

**Probe:**

- **If yes**,

How does your facility report the data to CARSS system?

What data (indicators) does your facility report to the system?

How is the quality of the data collected by your facility?

- **If no**,

Do you want to join the CARSS system? Why?

- 1. What is your understanding on current situation of AMR in China?
- Do you know any policies or regulations on combating AMR?

**Probe:**

Eg: *Status Report on Antimicrobial Administration and Antimicrobial Resistance in China*, surveillance system, etc.

- 1. Do you know who or which institution oversees administration on surveillance of AMR in China?

**Probe:**

- Eg: National Health Commission, etc.
- What are their main duties/functions?
  1. Do you have pharmacy administration committee in your facility?
- **If yes**,

What are its main duties?

How many staff in the committee and their background?

- 1. Do you have laboratories in your health facility?
- **If yes**,

Do you have antimicrobial susceptibility test in your health facility?

Can you specify the detailed procedure?

- **If no**, how to solve the AMR problem for a patient?
  1. Can you specify the detailed procedure on surveillance of AMR in your health facility?

**Probe:**

- Reporting system, surveillance system, etc.
  1. What kinds of training has been provided to the health professionals?

**Probe:**

- the training format: lecture/exam/conference on AMR, etc.
- the frequency of these trainings
  1. What are the challenges in combating AMR in your facility?
  2. Do you have any suggestions on improving current measurements of surveillance of AMR?

**Probe:**

- System/ training, etc.

1. **Impact of Covid-19**
   1. What was the impact of Covid-19 on your facility?

- outpatient / inpatient services, management, etc.
- what was the reaction of Fever Clinic and Infection Dept. of your facility?
  1. What was the impact of Covid-19 on antimicrobial drug use?
- the antimicrobial drug use condition compared to usual situation

(has preventive antimicrobial drug prescription increased?)

- What was the management of antimicrobial drugs in your facility during the outbreak (compared to usual)?

1. **Interview guidelines with doctors in health institution**

**(departments/divisions with higher possibilities on prescribing AB:**

**Respiratory, internal physicians, gastroenterology and so on. The doctors should have more experience but he/she is not the dean of the department, also will balance the gender)**

1. **Basic information**
   1. Could you make a self-introduction?

**Probe:**

- age, gender, educational level
- department/division, administrative post, clinical speciality, professional ranks and titles, length of service
  1. Could you introduce your daily work?

**Probe:**

- clinical services: how many patients per day?
- surgical services: how many patients per day/week? What is the normal practice on pre-/post-op treatment?

1. **usage of AB**
   1. What do you normally prescribe for common diseases, such as cold/diarrhea/ respiratory infection/alimentary infection, etc.?

- What kind of disease do you usually prescribe AB drugs?
- Please identify the types and names of the most commonly prescribed AB.
- Why you choose them?

**Probe:**

eg: for patient’s condition or AB’s safety/effective/quality/the type of the pathogen/whether it has AMR/the price/ whether the patient can afford/ drug compliance, etc.

- 1. Under what circumstances would you consider prescribing AB and the reasons for making those prescriptions?

**Probe:**

- Please identify the indications that you think antibiotics should/shouldn’t be prescribed.
- Reasons: your own experience/experience from senior doctors/ experience from colleagues/medication guides/textbook/literatures/learned from prescription seminar/the information provided by drug company, etc.
- Do you ever feel pressure from patients (peer physicians, regulators, others) to (or not to) diagnose or prescribe in a certain way?
  1. Have you ever experienced the situation that you think AB is not needed but the patient asks for AB?

**Probe:**

- **If so**, how often?
- Could you describe some of the cases?
  1. Roughly speaking, could you estimate these percentage of the following points?
- proportion of AB in your prescriptions for inpatient?
- proportion of AB in your prescriptions for outpatient?
- proportion of AB prescribing in your department for inpatient?
- proportion of AB prescribing in your department for outpatient?
  1. Has your patient or any patient from your department had episode of adverse reaction after taking AB?

**Probe:**

- How does this affect your prescription on antibiotics for your patients?
  1. How do you explain how to use AB to your patients?

Eg: What did you talk to the last patient about usage of AB?

1. **Surveillance of AMR**
   1. Do you know the current situation of AMR in China?
   2. Do you know the specific policies or regulations on administration on usage of AB and surveillance of AMR in China?

**Probe:**

- **if so**, what do you think of the scientific and operational characters of them?
- how does your hospital’s performance?
- have you ever taken any related training?

**Probe:**

- the format: lecture/exam/conference? the frequency?
  1. What do you think is the root cause of the irrational use of AB?

**Probe:**

- lack of knowledge/ prescription norms learned from senior doctors/need extra income to compensate the low salary/ patients’ expectation and stress/ over-marketing of pharmaceutical companies, etc.
  1. What do you think about the issue of AMR? Any suggestions?

1. **Surveillance of AMR**
   1. What was the impact of Covid-19 on your daily work?

- The number of outpatient / inpatient services, etc.
  1. Has Covid-19 have any impact on your drug prescription?
- the antimicrobial drug prescription compared to usual situation

(has preventive antimicrobial drug prescription increased?)

- Has the management of antimicrobial drugs in your facility changed during the outbreak (compared to usual)?

1. **Interview guidelines with pharmacists in health institution**

**(pharmacy department/ labs)**

1. **Basic information**

1.1 Could you make a self-introduction?

**Probe:**

- age, gender, educational level
- department/division, administrative post, clinical speciality, professional ranks and titles, length of service
  1. Could you introduce your daily work?

1. **The usage of AB situation**
   1. What do you know about the usage of AB situation in your health facility?

**Probe:**

- how many prescriptions in your facility per day?
- how many AB prescriptions in your facility per day?
- how many kinds of AB drugs in your pharmacy? Please give the **exact number** and list some of their names (or kinds).
  1. Roughly speaking, could you estimate these percentage of the following points?
- proportion of AB prescriptions for inpatient in your facility?
- proportion of AB prescriptions for outpatient in your facility?
  1. How about the usage of AB among different department in your health facility?

**Probe:**

- prescription for treatment & prevention
- the most and the least usage department
- please identify the types and names of the most commonly prescribed AB.
  1. Do you know who or which institution oversees administration on usage of AB?

**Probe:**

- Eg: Department of Hospital Service/Department of Drug and Food at Ministry of Health, etc.
- What are their main duties/functions?
  1. What do you know about the usage of AB management in your health facility? How do you involve in it?

**Probe:**

- Has your health facility designed any management goal or performance indicator regarding usage of AB?
- what’s the goal & indicator system & data source?
  1. What are the main regulations of administration on usage of AB in your health facility? How is your role?

**Probe:**

- **format:** management of antimicrobial drug catalogue, surveillance system, function of pharmacy staff, etc.
- Who **administrate** the usage of AB?
- Who **evaluate** the AB prescription?
- What do you think of the effectiveness of those regulations?
- Do you have pharmacy administration committee?
  - If yes,

What are its main duties?

how many staff in the committee and their background?

- 1. Can you specify the detailed procedure on surveillance of usage of AB in your health facility?

**Probe:**

- Reporting system/prescription review, etc.
- what information or indicators do you collect from each department in the system? (Eg: DDDs,etc.)
  1. Do you know any incentive or punishment mechanism in your facility on administrating doctors prescribing AB?

**Probe:**

- Eg: punish or fine the doctors who prescribed too much AB? the lower usage rate, the higher salary/deprivation of prescription?
  1. What kinds of training on AU have you provided to the health practitioners in your facility?

**Probe:**

- the training format: lecture/exam/conference on AU, etc.
- the frequency of these trainings
  1. Do you have laboratories in your health facility?
- **If yes**, do you have antimicrobial susceptibility test in your health facility?
- Can you specify the detailed procedure?
- **If no**, do you know how to solve the AMR problem for a patient?
  1. Has any patient in your health facility had episode of adverse reaction after taking AB?

**Probe:**

- How does this affect your management or distribution of antibiotics for the patients?
  1. Do you explain how to use AB to the patients normally?
  2. What do you think the root cause of the irrational use of AB?

**Probe:**

- lack of knowledge/ prescription habit learned from senior doctors/need extra income to compensate the low salary/ patients’ expectation and stress/ over-marketing of pharmaceutical companies
  1. What are the challenges in managing usage of AB in your facility?
  2. In your opinion, do other types of health policies cast any impacts on the administration and usage of AB in China?

**Probe:**

- Eg: medical insurance policy/ essential medicine policy
  1. Compared with other health facility at the same level, what do you think are the characteristics and innovations of the management of usage of AB in your facility?

**Probe:**

- Any successful experience?
  1. Do you have any suggestions on improving current measurements of administration on usage of AB in your facility?

**Probe:** System/ training, etc.

1. **Surveillance of AMR**
   1. Do you know the current situation of AMR in China?
   2. Do you know the specific policies or regulations on administration and surveillance of AMR in China?

**Probe:**

- **if so**, what do you think of the scientific and operational characters of them?
- how does your facility’s performance?
  1. have you ever taken any related trainings on AMR?

**Probe:**

- the training format: lecture/exam/conference on AU, etc.
- the frequency of these trainings
  1. What do you think about the issue of AMR in China? Any suggestions?

**4. Impact of COVID-19**

- 1. What was the impact of Covid-19 on your daily work?
- Workload changes, etc.
  1. What is the impact of Covid-19 on antimicrobial drug use and management (answered only by pharmacist)?
- what are the most frequently prescribed drugs by your facility during the outbreak? List the top five.
- the antimicrobial drug prescription compared to usual situation

(has preventive antimicrobial drug prescription increased?)

- has the management of antimicrobial drugs in your facility changed during the outbreak (compared to usual)?

1. **Interview guideline with patients at health facility**

**(Respiratory or internal outpatient clinic, with higher possibility using AB or be given AB)**

1. **Basic information**
   1. Could you make a self-introduction?

**Probe:**

- age, gender, educational level
- type of medical insurance
  1. Why did you come to the hospital today?

**Probe:**

- Is this your first visit to this facility in general/ first visit for your uncomfortable?
- Did you go to other health facilities before this visit?
- Why this hospital/doctor?
  1. Did you use AB drugs without doctor’s permission before this visit?
- If yes, how did you use them?
  1. What are your symptoms and for how long?
  2. How did the consultation go (details)?

**Probe:**

- Did the doctor make diagnosis or ordered any examinations?
- What did you get from the doctor (prescription and medication)?
  1. Do you know the name or type of AB medicine that you were given?
- (patient might not sure or know, we **might ask them to provide** the names of medication/prescription, if allowed and readable?)
  1. Did the doctor ask your idea or opinion on what treatment or medicine you would like to use?

**Probe:**

- Did the doctor explain to you why choose these drugs/treatment?
- Did the doctor give you instructions on how to use the drugs and what did he/she say exactly?
  1. Where are you going to purchase your drugs and why?

**Probe:**

- price, brand, package, pharmacy, medical insurance, etc.
- **if outside hospital**, does the pharmacist give any suggestions/ask for prescriptions?
- Did you or anybody you know ever purchased prescription drug without consulting a doctor in hospital (some pharmacy might hire doctors to give prescription over the counter)?
  1. How are you going to use the AB drugs?

**Probe:**

- Are you going to take all types of your drugs or only specific one?
- Did the doctor tell you how many times per day/when to stop using the drugs?
- When will you stop using the drugs?

**Probe:**

- follow the doctor’s instructions, the manual of drug, or after you finish the whole package?
  1. Do you take all the medicine or are you going to save some for the future?

**Probe:**

- What are you going to do with the remaining medicine (if any) after you stop taking it?

1. **Usage of AB**
   1. What do you know about AB?

**Probe:**

- How do you understand antibiotics?
- Where did you get this knowledge?
- How do you know on what way you should take the medicines?
  1. What do you do if you or your kids get a cold?

**Probe:**

- Eg: rest at home/use the drugs kept in home/go to the drugstore/ go to the doctor, etc.
  1. Have you bought AB in drugstore without prescription? Please describe the experience in detail.

**Probe:**

- **If have**, what kinds of AB do you usually buy or keep in your home?
  1. Under what circumstances have you used AB? Please describe the experience in detail.
  2. Would you expect doctor to prescribe you with AB for common diseases such as cold?
  3. Have you ever experienced the situation that you think AB is needed but the doctor thinks not? Please describe the experience in detail.
- **If yes**, what did you do? Buy AB in a drugstore?
  1. Do you or anybody you know have had episode of adverse reaction after taking AB? Please describe the experience in detail.

**Probe:**

- Do you or anybody you know suffer from allergies on antibiotics?
- If yes, any adverse reaction?
  1. Have you ever learnt about the rational usage of AB?

**Probe:**

- Under what circumstances do you think antibiotics should be prescribed/used? Why?
- Could you give some examples on how to safely and rationally use antibiotics?
- The way you learn: doctor/media/pharmacist/common sense/leaflet or poster from health facilities, etc.

1. **Surveillance of AMR**
   1. Have you ever learnt about the harm of AMR?

**Probe:**

- Do you know AMR?
- What cause it?
- Do you know how the hospital/government handle the situation?
- Do you know any policies on AMR?
- Where did you learn about this?
- Do you think it is relevant to you and your family?
- Any concerns or suggestions?
  1. Have you ever received public education on rational usage of AB? Exactly how?

**Probe:**

- advertisement/public classes, etc.

1. **Impact of Covid-19**
   1. What was the impact of Covid-19 on your medical visit or consultation?
   2. What is the impact of Covid-19 on your medication?

- did you or your family go to a hospital or drugstore to buy medicine during the outbreak? List drug name
- did you or your family take drugs to prevent or enhance resistance during the outbreak?
- Any antimicrobial drugs in the drugs above?

1. **Interview guidelines with drugstore staff**
2. Could you introduce yourself? (age, gender, education, major, length of service)
3. Could you introduce the basic information of your drugstore?

Probe:

- staff background information? What’s your daily work like?
- Scale of your drugstore? The types and quantity of drugs in your drugstore?
- Is there any online drug sale service in your drugstore? What is the process of purchasing medicine online?

1. Could you introduce the sales of drugs in your drugstore?

Probe:

- name and type of the most purchased drug in your drugstore?
- what are the monthly sales of online and offline drugs?
- what is the daily number of people who come to the store to buy medicine?
- what is the daily number of people who buy medicine in your online store?

1. Do you have any antibiotics in your pharmacy?

Probe:

- How many kinds? Can you provide the list of those medicines?
- what is the monthly sales volume of antibiotics? Can you provide the sales list?
- the most commonly purchased antibiotics and their names?

1. Can people buy antibiotics directly in your store?

Probe:

- if not, how can people buy antibiotics in your store?
- is there any difference between buying antibiotics online / offline in your store?

1. Has your drugstore hired an attending doctor?

Probe:

- If so, what’s the basic info of the doctor? Age, major, length of work?
- what are the daily work of the attending doctor?
- can the attending doctor prescribe antibiotics for customers?

7. What’s the impact of Covid-19 on your drugstore?

Probe:

- has customer visits and drug sales changed at your store? How?
- has the changes in the sales of antibiotics changed? How?

1. **Interview guidelines with policy-makers**

**(Experts from Department of Hospital Service, Department of Drug and Food at Ministry of Health, AMR Working Group, Universities and other related institutions)**

1. **Basic information**
   1. Could you make a self-introduction?

**Probe:**

- age, gender, educational level
- department, administrative post, clinical speciality, professional ranks and titles, length of service (length of service of current position)

1. **usage of AB part**
   1. What is your understanding on current situation of usage of AB in China?
   2. What is your understanding on the national or local strategies to control usage of AB?

- are they scientific/operational/effective/efficient?
  1. What is your consideration when design/implement a policy or a plan of administration on usage of AB?
  2. In your opinion, what are the challenges or barriers for the current administration on usage of AB?
  3. Do you have any suggestions on improving current measurements of administration on usage of AB in China?

1. **AMR part**
   1. What is your understanding on current situation of AMR in China?
   2. What is your understanding on the national or local strategies to monitor or combat AMR?

- are they scientific/operational/effective/efficient?
  1. What is your consideration when design/implement a policy or a plan of monitoring AMR?
  2. In your opinion, what are the challenges or barriers for the current surveillance of AMR?
  3. Do you have any suggestions on improving surveillance of AMR in China?

**Other Sectors**

1. **Interview guideline with administrator in local Medical Products Administration office/local Health Committee**
2. Could you make a self-introduction?

**Probe:**

- age, gender, educational level
- department/division, administrative post, clinical speciality, professional ranks and titles, length of service

1. Please introduce the situation on usage of antibiotics in this area)?
2. How many of the top ten drugs are the antibiotics?

Probe:

- Top ten in hospitals (public and private)
- Top ten in drugstores

1. Do you know the national policy on administration of antibiotics and guideline on usage of antibiotics?

- How does the policy or guideline relate to your department’s administration on food and drugs?

1. What kinds of administrative measurement does your facility applied on the usage of antibiotics?

**Probe:**

- Any management goals or performance indicators set up for the subordinate agencies or facilities?
- How about the effectiveness?
- Any incentives for better or punishment for violation? Please give some examples in detail.

1. How does your institution supervise on production and sales on antibiotics?

**Probe:**

- Can you specify the procedure of the supervision?
- How about the effectiveness?

1. Has your institution set up surveillance system on the usage of any antibiotics?

- Eg: for medical facilities, pharmacy, fish breeding and poultry, manufacturing enterprise?
- any surveillance for online sale?

1. How about your management team and division of duties on administration of antibiotics?

**Probe:**

- Eg: Human resources, financial support, authority of administrative penalties (and related regulations, effects)
- Any professional trainings for them? If yes, please introduce in details.

1. Has your institution provided any public campaign or education on rational usage of antibiotics to employees from related industry or residents? Please provide the details.

**Probe:**

- What kind of campaign?
- The content and forms.

1. Has your institution cooperated with other agencies on managing the problem of irrational usage of antibiotics? Please provide the details.

- public agencies/private agencies.
- How did you cooperate with them?
- What are you in charge of exactly?

1. What are your institution’s experience and challenge on managing irrational usage of antibiotics?

- Any good experiences?
- You’ve come across what practical challenge?

1. How do you think about AMR in China?

- Any suggestions on how to improve current antibiotics administration and surveillance on AMR?

1. What are your institution’s experience and challenge on combating AMR?

- Any good experiences?
- You’ve come across what practical challenge?

1. The impact of Covid-19 on local drug use and management?

- the general condition of local antimicrobial drug use during the outbreak?
- has the local management of antimicrobial drugs changed during the outbreak (compared to usual)?

1. **Interview guideline with administrator from Ecology and Environment Bureau department**
2. Could you make a self-introduction?

**Probe:**

- age, gender, educational level
- department/division, administrative post, clinical speciality, professional ranks and titles, length of service

1. Please introduce the situation of local antibiotics contamination and emission?
2. Do you know the related national policy and law on environmental surveillance on antibiotics contaminant?

- Eg: The *Work Division Scheme for National Action Plan to Contain Antimicrobial Resistance(2016-2020)*, etc.

1. What kinds of environmental safety evaluation system and standard does your department set up on antibiotics?

**Probe:**

- Eg: pharmaceutical company/ Biotechnology company, etc.

1. Has your department set up any environmental monitoring and governance measurements on antibiotics?

**Probe:**

- Any goals or indicators?
- How about the effectiveness?
- Any incentives for better or punishment for violation? Please give some examples in detail.

1. How about your management team and division of duties on administration of antibiotics?

**Probe:**

- Eg: Human resources, financial support, authority of administrative penalties (and related regulations, effects)
- Any professional labs or auditing department?
- Any professional trainings for them? If yes, please introduce in details.

1. Has your institution provided any public campaign or education on rational usage of antibiotics to employees from related industry or residents? Please provide the details.

**Probe:**

- What kind of campaign?
- The content and forms.

1. Has your institution cooperated with other agencies on managing the problem of irrational usage of antibiotics? Please provide the details.

**Probe:**

- public agencies/private agencies.
- How did you cooperate with them?
- What are you in charge of exactly?

1. What are your institution’s experience and challenge on managing irrational usage of antibiotics?

- Any good experiences?
- You’ve come across what practical challenge?

1. How do you think about AMR in China?

- Any suggestions on how to improve current antibiotics administration and surveillance on AMR?

1. What are your institution’s experience and challenge on combating AMR?

- Any good experiences?
- You’ve come across what practical challenge?

1. **Interview guideline with administrator from Bureau of Agriculture and Rural Affairs ( animal husbandry department)**
2. Could you make a self-introduction?

**Probe:**

- age, gender, educational level
- department/division, administrative post, clinical speciality, professional ranks and titles, length of service

1. Please introduce the situation of local veterinary antibiotics usage/ antibiotics usage on animal.
2. Do you know the related national policy and guideline on veterinary antibiotics usage/antibiotics usage on animal?

**Probe:**

- Eg: *Regulations on the Administration of Veterinary Drugs*, QR-code on animals, etc.

1. What kinds of management procedure does your department set up on veterinary antibiotics?

**Probe:**

- Any goals or indicators?
- How about the effectiveness?
- Any incentives for better or punishment for violation? Please give some examples in detail.

1. Has your department set up any surveillance platform on veterinary antibiotics?

- Has your department set up a surveillance network on production and operation of major fishery/poultry enterprise?

**If yes**, how does if oprate?

- Any indicators of surveillance?
- Do you have a reporting system to superior institution?

1. How about your management team and division of duties on administration of veterinary antibiotics?

**Probe:**

- Eg: Human resources, financial support, authority of administrative penalties (and related regulations, effects)
- Any professional labs or auditing department?
- Any professional trainings for them? If yes, please introduce in details.

1. Has your institution provided any public campaign or education on rational usage of veterinary antibiotics to employees from related poultry industry? Please provide the details.

**Probe:**

- What kind of campaign?
- The content and forms.

1. Has your institution cooperated with other agencies on managing the problem of irrational usage of veterinary antibiotics? Please provide the details.

**Probe:**

- public agencies/private agencies.
- How did you cooperate with them?
- What are you in charge of exactly?

1. What are your institution’s experience and challenge on managing irrational usage of veterinary antibiotics?

- Any good experiences?
- You’ve come across what practical challenge?

1. How do you think about AMR on animal husbandry and veterinary in China?

- Any suggestions on how to improve current veterinary antibiotics administration and surveillance on AMR?

1. What are your institution’s experience and challenge on combating AMR?

- Any good experiences?
- You’ve come across what practical challenge?

1. The impact of Covid-19 on local veterinary antibiotics use and management?

- the general condition of local veterinary antibiotics use during the outbreak?
- has the local management of veterinary antibiotics changed during the outbreak (compared to usual)?

**Personals who use veterinary antibiotics**

1. **Fishery/poultry industry practitioners**
2. Could you make a self-introduction?

**Probe:**

- age, gender, educational level

1. What kind of animal do you raise?

**Probe:**

- Size of your farm?
- length of service?

1. Under what circumstances would you use antibiotics on poultry and livestock?

**Probe:**

- For which types of indications do you think ABs should be used?

Eg: disease happen/ prevent disease/ promote growth, etc.

- During the past year, how many times did you use AB on animals?

1. Does the local authority provide support or instruction on the veterinary usage of drugs?

**Probe:**

- Any trainings?
- The content and forms?

1. How do you normally use fodders with veterinary antibiotics?

**Probe:**

- types, amounts and frequencies?
- How do you control the dosage?

1. What is your normal choice of fodder?

**Probe:**

- Why do you choose the brand?
- Do you know its ingredients? (Can you give us some sample of it?)
- Why it is your best choice?
- Does it help to prevent diseases?

1. Do you have any special fodders, additives, or medicines for the animals?

- If yes, please provide details.
- Have you ever used human medicines on the farm/ranch/etc.?

1. What types of channel do you have on purchasing antibiotics and fodder additives?

- Eg: local veterinary drugstores/ veterinary clinics, etc.

1. Do you know the government policy and regulation on antibiotics usage in poultry and livestock breeding?

**Probe:**

- Eg: QR-code traceability for the use of veterinary drugs/ plan for reduced use of veterinary antimicrobials, etc.
- Do you know the punitive measures for violation behaviour?

1. How do you think about the inspection and supervision measures of supervision department on antibiotics usage?

**Probe:**

- How about the effectiveness?
- How does the supervision and governance effects your poultry and livestock breeding behavior?

1. Have you ever taken any public campaign or education on rational usage of antibiotics?

**Probe:**

- Any trainings?
- The content and forms?

1. Do you use AB on your livestock?

**Probe:**

- If yes, would you consume your products?
- Would you let you family and children consume your products?
- Do you have family livestock that reserved only for yourself and your families?

1. Do you have any experience or tricks on picking fish/poultry for food?
2. Do you know the harm caused by abusive usage of antibiotics in poultry and livestock breeding, and its harmful effect on human health?

- Through what channel did you learn?

1. How do you think about using antibiotics in poultry and livestock breeding in the future?

- Any suggestions?

1. What was the impact of Covid-19 on your use of veterinary antibiotics?

- has the use of preventive medicine increased?
